# The German version of the highly sensitive child scale: Psychometric properties and identification of sensitivity groups

**DOI:** 10.1007/s12144-026-09244-w

**Published:** 2026-03-23

**Authors:** Susanne Wehrli, Jana Hochreuter, David Buttelmann, Francesca Lionetti, Michael Pluess, Niamh Oeri, Helen Koechlin

**Affiliations:** 1 Department of Psychosomatics and Psychiatry, University Children’s Hospital, University of Zurich, Zurich, Switzerland; 2 Division of Child and Adolescent Health Psychology, Department of Psychology, University of Zurich, Zurich, Switzerland; 3 Children’s Research Centre, University Children’s Hospital Zurich, University of Zurich, Zurich, Switzerland; 4 University Research Priority Program “ITINERARE –Innovative Therapies in Rare Diseases”, University of Zurich, Zurich, Switzerland; 5 Institute for Psychology, Developmental Psychology, University of Bern, Bern, Switzerland; 6 Department of Neuroscience, Imaging and Clinical Sciences, G. d’Annunzio University of Chieti-Pescara, Chieti, Italy; 7 School of Psychology, University of Surrey, Guildford, UK; 8 Department of Biological and Experimental Psychology, Queen Mary University, London, UK

**Keywords:** Children and Adolescents, Environmental Sensitivity, Highly Sensitive Child scale, Self-report, Parent report

## Abstract

Individuals differ in their sensitivity to external stimuli. The Highly Sensitive Child (HSC) scale can be used to measure sensitivity in children and adolescents. However, the German version has yet to be validated. We examined the psychometric properties of the German self- and the parent report version of the HSC. Measurement invariance (MI) across age groups was tested for the parent report version and latent profile analysis (LPA) was used to identify sensitivity groups. Pooled data from German-speaking countries (*N*= 250 self-reports; *N*= 3657 parent reports) was included. The factor structure was evaluated using confirmatory factor analysis (CFA), reliability was quantified by Cronbach’s alpha (α) and MI was assessed by multigroup CFA using the optimal model. An exploratory analysis was carried out across age groups. CFA indicated the bifactorial model as the best fit for the HSC. Reliability of the total score across age groups was acceptable, but the MI was not. In the LPA, we found two sensitivity classes for early, middle childhood, and adolescent parent-reports, and four groups for self-report. The German version of the HSC seems to be a valid measurement of sensitivity. However, future research should examine age differences across childhood more closely.

**Supplementary information:**

The online version contains supplementary material available at 10.1007/s12144-026-09244-w.

Individuals differ in their sensitivity to environmental conditions, in that some are more affected by their surroundings than others (Pluess, 2015). Even though much research on Environmental Sensitivity focuses on adults, this capacity is also documented in several studies featuring children and adolescents (Belsky and Pluess 2013; Ellis et al. 2011a, b; Obradović et al. 2010; Pluess 2015). To date, the Highly Sensitive Child Scale (HSC) is the dominant sensitivity measure that can be used in children and adolescents (Pluess et al., 2018). Self-report questionnaires are easy and inexpensive to administer in research and clinical settings, but require a systematic examination of their measurement properties (Aithal & Aithal, 2020). While the English version has undergone extensive psychometric validation (Pluess et al., 2018), the German version of the HSC has yet to be validated.

## Environmental sensitivity

In 2015, an overarching meta-framework called Environmental Sensitivity was introduced to incorporate several theories that examine differences in the ability to register and process external stimuli (Pluess, 2015). Among these, the Diathesis-Stress Model has been widely recognized for its explanation of individual variability in response to adverse life events (Monroe & Simons, 1991). In contrast, the framework called Vantage Sensitivity, a more recent model derived from Differential Susceptibility (Pluess & Belsky, 2013), states that some children are more responsive to positive experiences, such as psychological interventions (Albert et al., 2015; Pluess, 2017). Several theoretical frameworks, including Sensory Processing Sensitivity (SPS; Aron et al., 2012; Aron & Aron, 1997), Biological Sensitivity to Context (BSC; Boyce & Ellis, 2005), and Differential Susceptibility (DS; Belsky, 1997; Belsky & Pluess, 2009a, b) each provide unique insights into the construct of Environmental Sensitivity (Ellis et al. 2011a, b; Pluess 2015). Collectively, however, these models agree on the notion that more sensitive individuals differ from less sensitive peers not only in their response to environmental adversity, but also in response to positive aspects of their environment (such as social support or sensitive parenting). The focus of the present study lies on SPS. SPS describes a genetically influenced, relatively stable personality trait that manifests in deeper cognitive processing of stimuli, a lower threshold to sensory inputs, and high emotional reactivity (Aron et al., 2005, 2012; Aron & Aron, 1997). The Highly Sensitivity Person (HSP) scale (Aron & Aron, 1997) was originally developed to assess SPS in adults.

## The highly sensitive child scale

In 2018, the HSC was developed and validated, based on the HSP scale (Pluess et al., 2018). This 12-item scale measures SPS in children from the age of 8 years onwards and reflects the three dimensions of the HSP scale: Ease of Excitation (EOE), Low Sensory Threshold (LST), and Aesthetic Sensitivity (AES) (Pluess et al., 2018). The scale is available as self-report (Pluess et al., 2018) and parent report (HSC-PR; Sperati et al., 2022). Measured with the HSC, sensitivity is a continuously interpreted trait. While variable-centered research approaches indicate a normal distribution in a healthy population, person-centered approaches demonstrate that sensitivity can be categorized into three groups: low, medium and high sensitivity (Pluess et al., 2018). The original SPS theory was based on sensitivity as a unidimensional construct with an overall sensitivity factor (Aron & Aron, 1997). However, the factor structure of the SPS construct was unclear in subsequent studies (Greven et al., 2019; Konrad & Herzberg, 2017). More recent studies have suggested a three-factor model with the factors EOE, LST, and AES (e.g. Smolewska et al., 2006). The exploratory and confirmatory factor analyses by Pluess et al. (2018) revealed a bifactor solution to be a better fit for the HSC than the one- or three-factor model. This means that the variance in the HSC items can be explained by both a general underlying dimension (Environmental Sensitivity) and the three dimensions of the subscales (EOE, LST, and AES) (Pluess et al., 2018). These findings were replicated in Dutch, Belgian, and Chinese validation studies of the HSC (Dong et al., 2022; Liu et al., 2023; Weyn et al., 2021, 2022) and also for the HSC-PR (Sperati et al., 2022). However, the German versions of the HSC and the HSC-PR have not been validated yet. For the HSC, two studies tested measurement invariance (MI) for separate age groups to examine differences across age: Early adolescents versus middle to late adolescents (Weyn et al., 2021, 2022) and elementary versus middle school children (Liu et al., 2023). Preschoolers and school-age children up to pre-adolescence were the defined age groups for the HSC-PR (Sperati et al., 2022). Measurement was partially invariant across the two age groups in all studies, while full configural invariance and at least partial metric and partial scalar invariance across age groups was evident (Liu et al. 2023; Sperati et al. 2022; Weyn et al. 2021, 2022).

## The present study

The present study aims to examine the psychometric properties of the German translation of the HSC in German-speaking samples with data from Switzerland, Germany, and Austria. Factor structure, internal consistency and dimensionality will be assessed across age groups for both scale versions, the self-report and the parent report. The MI will be computed for the parent report version. Additionally, LPA will be used to test for the existence of sensitivity groups. Based on the original study (Pluess et al., 2018) and translated versions in other languages (Dong et al. 2022; Liu et al. 2023; Weyn et al. 2021, 2022), we expect a bifactorial model (i.e., with one general sensitivity factor and three subdimensions) to fit the data better than a one-factor model (i.e., general sensitivity factor) or a three-factor model (i.e., the subscales as factors). Further, three distinct latent classes are expected: A low, medium, and high sensitivity class.

## Methods

### Participants and procedure

Data was pooled from several studies conducted in Switzerland, Germany, and Austria, including a parent report study of preschool children, an experimental self-report study of healthy adolescents, a self-report online survey of adolescents with chronic pain, and a self-selected cohort derived from parent report online questionnaires. The collective dataset comprised 3907 participants, subdivided into 250 adolescent self-report and 3657 parent report subsets. These subsets are designated for separate analyses according to the mode of data collection, namely self-report and parent report.

For the collection of parent-reported data on preschool children, recruitment was carried out through public kindergartens and nurseries. Recruitment for parent-reported data on children and adolescents was facilitated through the Sensitivity Research website (https://sensitivityresearch.com/de/selbsttests/test-fur-eltern-und-kinder-8-18-jahre/). Prior to participation, informed consent was obtained from parents either in writing or digitally. With regard to self-reported data, adolescents with chronic pain were identified and recruited online through a combination of chronic pain patient forums, educational institutions, specialized pain treatment centers, and promotion on social media platforms. Conversely, healthy adolescents and young adults were recruited through local educational institutions and promotional material posted on the official website of the University of Basel. Written informed consent was obtained from adolescents prior to their participation in each study.

The hypotheses, research questions, measures, and analysis strategy were preregistered via the Open Science Framework (https://archive.org/details/osf-registrations-53bnt-v1).

### Measures

Environmental Sensitivity: Sensitivity was assessed with either the self-report or the parent report version of the Highly Sensitive Child scale (HSC and HSC-PR; Pluess et al., 2018; Sperati et al., 2022). The scale consists of 12 items. Items are rated on a seven-point Likert scale ranging from *not at all true* (1) to *extremely true* (7). Higher scores indicate higher sensitivity to the environment. The Ease of Excitation (EOE) subscale consists of 5 items (e.g., “I get nervous when I have to do a lot in little time”), the Aesthetic Sensitivity (AES) subscale contains 4 items (e.g., “I love nice smells”), and the Low Sensory Threshold (LST) subscales includes 3 items (e.g., “Loud noises make me feel uncomfortable”). The self-report items are identical to the parent version, with the only difference being that the “I” in the self-report version is replaced by “my child” in the parent version. The self-report and parent-report versions of the scale were translated using the standardized translation protocol implemented by the authors of the original English scale, namely Pluess et al. (2018). This procedure involved forward, and backward translation as well as a consensus meeting carried out by native speakers and one of the authors (H.K.) to ensure linguistic and conceptual equivalence.

### Statistical analysis

Analytical procedures were performed using R statistical software (version 4.2.3; R Core Team, 2023). The missMDA (Josse & Husson, 2016) and FactoMineR (Lê et al., 2008) packages, which perform multiple imputation via iterative principal component analysis, were used to handle missing data. Before proceeding with imputation, we first examined the pattern of missing data in the parent report sample using Little’s MCAR test to determine whether the data were missing completely at random (MCAR), which was confirmed (Little, 1988).

CFA was conducted within a structural equation modeling (SEM) framework using the lavaan package (Rosseel, 2012). This analysis aimed to assess the factor structure utilizing the one-factor solution as originally proposed by Aron and Aron (1997), the three-factor solution and the bifactorial model (Pluess et al., 2018). For the bifactorial model of the parent report, two models were evaluated: the original version and one without item 7. Item 7 states “my child doesn’t like watching TV programs that have a lot of violence in them”, which for younger children (i.e., under the age of 7 years) might not apply, as parents probably control more strictly what their young children watch. We therefore omitted item 7 in the analyses of the parent report version. For the model in which item 7 was omitted, the factor loadings for the two existing items of the LST subscale (items 2 and 11) were constrained to 1.0 to ensure measurement equivalence. Model selection was based on the chi-squared difference test. It is not possible to directly “compare” non-nested models using standard statistical tests (such as the chi-square difference test); however, these models can still be “evaluated” based on their fit indices and conceptual justification. Therefore, we avoid using the term “comparison” in this context and instead refer to it as an “evaluation” to determine which model has better fit indices or aligns more closely with theoretical expectations, rather than conducting a formal statistical comparison. The goodness of fit of the selected model was assessed using various indices including the Comparative Fit Index (CFI), the Relative Noncentrality Index (RNI), the Root Mean Square Error of Approximation (RMSEA) and the Standardized Root Mean Square Residual (SRMR) (Hu & Bentler, 1999). Acceptability thresholds were set at CFI and RNI values ≥ 0.95 for a good fit and ≥ 0.90 for an acceptable fit; RMSEA values < 0.05 indicated a good fit and < 0.08 an acceptable fit; SRMR values < 0.50 indicated a good fit and < 1.00 an acceptable fit. Reliability was quantified by Cronbach’s alpha (α) determined using the psych package (Revelle, 2024), with α ≥ 0.9 indicating excellent reliability, ≥ 0.8 good and ≥ 0.7 acceptable (George & Mallery, 1999). In addition, we calculated McDonald’s omega (ω) as a reliability indicator, with values of 0.70 or higher considered satisfactory (Viladrich et al., 2017).

MI across different age cohorts was subsequently assessed by multigroup CFA using the optimal model. Configural, metric, scalar, and uniqueness invariance were examined using the same fit indices as in the selected CFA. Age cohorts were divided into early childhood (7 years or younger), middle childhood (8 to 13 years), and adolescence (14 to 19 years) (Kail, 2016).

LPA was performed using the TidyLPA package (Rosenberg et al., 2018) based on the item level of the HSC. Models with different variances and covariances were estimated, comparing solutions from 1 to 6 classes using fit indices such as entropy, log-likelihood, Akaike information criterion (AIC), adjusted Bayesian information criterion (aBIC), Bayesian information criterion (BIC), consistent Akaike information criterion (CAIC), Lo-Mendell-Rubin likelihood ratio test (LMR) and bootstrapped likelihood ratio test (BLRT) (Ferguson et al., 2019). A superior model fit was indicated by lower AIC, aBIC, BIC and CAIC values, while an entropy value ≥ 0.80 indicated minimal classification uncertainty. The LMR and BLRT were used to assess model fit and complexity.

## Results

### Participant characteristics

Our sample included a total of 3657 parent reports with a child mean age of 8.5 years (SD = 3.4), and 250 self-reports with a mean age of adolescents of 17.56 years (SD = 1.08), with a close to balanced gender ratio. Table 1 presents descriptive statistics for the parent report and adolescent self-report samples.

**Table 1:** Descriptive Statistics of Samples

Parent report	*N* = 3657
Age, *M* (*SD)*	8.5 (3.4)
Gender, *n* (%)	
Male	1,828 (50%)
Female	1,815 (50%)
Prefer not to say	8.5 (3.4)
HSC	
Total mean, *M* (*SD)*	5.42 (0.85)
AES mean, *M* (*SD)*	5.47 (1.07)
EOE mean, *M* (*SD)*	5.35 (1.07)
LST mean, *M* (*SD)*	5.48 (1.16)
Adolescent self-report	*N* = 250
Age, *M* (*range)*	17.56 (1.08)
Gender, *n* (%)	
Male	122 (49%)
Female	128 (51%)
HSC	
Total mean, *M* (*SD)*	4.62 (0.82)
AES mean, *M* (*SD)*	5.32 (1.03)
EOE mean, *M* (*SD)*	4.33 (1.16)
LST mean, *M* (*SD)*	4.15 (1.44)

*AES * aesthetic sensitivity, *EOE * ease of excitation, *HSC * Highly Sensitive Child Scale, *LSE * low sensory threshold, *M* mean, *N* sample size, *SD* standard deviation

Overall, before imputation in the parent report sample for the HSC items, 119 out of 43,884 possible data points were missing, resulting in a missing data proportion of 0.27%. In the adolescent self-report 0 missing datapoints were detected. No outliers were removed to preserve the heterogeneity of the samples.

### Confirmatory factor analysis – global goodness of fit

To assess the factor structure, CFA was conducted on all three hypothesized theoretical models: the unidimensional model, the three-factor model, and the bifactorial model. A detailed overview of the factor loadings can be found in Supplementary Tables 1 and 2. Chi-squared test results indicated that the bifactorial model provided the best fit for both parent reports and adolescent self-reports. Consequently, the bifactorial models were selected for subsequent analyses, as detailed in Table 2.

**Table 2:** Model Evaluation: Chi-Squared Test for the One-Factor, Three-Factor, and the Bifactorial Models for Parent Report, Parent Report Separately for Age-Groups, and Adolescent Self-Report

Model	df	X^2^	X^2^ difference	*p*	AIC	BIC
HSC parent report (*N* = 3657)						
Bifactorial model	44	1782.0			150,236	150,521
Three-factor model	51	1974.3	152.29	< 0.001	150,414	150,656
One-factor model	54	4815.6	1395.76	< 0.001	153,250	153,473
HSC parent report early childhood group (aged 7 or younger) (*N* = 1511)						
Bifactorial model	44	678.11			62,555	62,800
Three-factor model	51	1118.94	343.79	< 0.001	62,982	63,190
One-factor model	54	1621.31	323.41	< 0.001	63,479	63,670
HSC parent report classes of the middle childhood group (aged 8 up to 13) (*N* = 1776)						
Bifactorial model	45	1037.39			71,492	71,738
Three-factor model	51	909.82	−91.51	1	71,352	71,566
One-factor model	54	2609.17	1174.27	< 0.001	73,045	73,243
HSC parent report adolescent group (aged 14 up to 19) (*N* = 370)						
Bifactorial model	45	252.64			14,475	14,651
Three-factor model	51	183.31	−65.425	1	14,393	14,546
One-factor model	54	587.56			14,792	14,932
HSC adolescent self-report (*N* = 250)						
Bifactorial model	42	173.17			11,060	11,229
Three-factor model	51	300.68	194.704	< 0.001	11,170	11,307
One-factor model	54	489.94	80.099	< 0.001	11,353	11,480

*df * degrees of freedom, *AIC * Akaike information criterion, *BIC * Bayesian information criterion, *X*^*2*^ chi-squared statistic

### Confirmatory factor analysis – local goodness of fit

An evaluation of the model fit metrics revealed that the CFI for the adolescent self-report models (CFI = 0.841) approached, but did not reach, the threshold for acceptable fit. The CFI was higher for the parent report, in particular exceeding the threshold for the variant excluding item 7 (see Table 3). The RMSEA for both the self-report and the parent report with and without item 7 was above 0.080, indicating suboptimal fit. The SRMR remained below 1.00 in all configurations, except for the adolescent self-report thus indicating suboptimal fit. Meanwhile, the RNI for each model variant was above 0.80, indicating satisfactory fit.

**Table 3:** Model Fit Indices for Bifactorial Models (Parent Report, Parent Report by Age Group, and Adolescent Self-Report) and Measurement Invariance Models Across Age Groups

Model	RMSEA	SRMR	CFI	RNI
HSC parent report (*N* = 3657)				
Bifactorial model	0.096	0.072	0.859	0.859
Bifactorial model, item 7 removed	0.095	0.085	0.882	0.882
HSC parent report early childhood group (aged 7 or younger) (*N* = 1511)				
Bifactorial model	0.090	0.069	0.881	0.881
Bifactorial model, item 7 removed	0.093	0.073	0.889	0.889
HSC parent report classes of the middle childhood group (aged 8 up to 13) (*N* = 1776)				
Three-factor model	0.089	0.078	0.844	0.844
Three-factor model, item 7 removed	0.089	0.065	0.867	0.867
HSC parent report adolescent group (aged 14 up to 19) (*N* = 370)				
Three-factor model	0.076	0.075	0.901	0.901
Three-factor model, item 7 removed	0.078	0.075	0.909	0.909
HSC adolescent self-report (*N* = 250)				
Bifactorial model	0.107	0.110	0.841	0.841
HSC parent report (*N* = 3657)				
Configural invariance model	0.151	0.103	0.556	0.556
Metric invariance model	0.138	0.123	0.579	0.579
Scalar invariance model	0.138	0.132	0.528	0.528

*CFI * Comparative fit index, *RNI * Relative noncentrality index, *RMSEA * Root mean square error of approximation, *SRMR * Standard root mean square residual

### Internal consistency

To assess the reliability of the HSC, Cronbach’s alpha coefficients were calculated for both the total score and each of the subscales. The results are presented in Table 4. In accordance with the criteria established by George and Mallery (1999), all the total scores were considered to be within the acceptable range of reliability, except for the adolescent self-report, which was marginally below the threshold. However, for both the parent report and the adolescent self-report, the AES, EOE, and LST subscales did not meet the predetermined acceptable reliability threshold, except for the EOE subscale of the parent report. The reliability of the LST subscale of the parent report was improved to a level considered good by excluding item 7. Concerning McDonald’s omega all values were considered satisfactory, except for the AES and EOE subscales for the adolescent self-report.

**Table 4:** Cronbach’s Alpha and McDonald’s Omega of HSC Total Scores and Subscales for the Parent Report, Parent Report Separately for Age-Groups, and Adolescent Self-Report

Latent Factor	α	ω
HSC parent report (*N* = 3657)		
Total	0.78	0.84
Total, item 7 removed	0.78	0.84
AES	0.66	0.70
EOE	0.71	0.72
LST	0.63	0.71
LST, item 7 removed	0.84	0.84
HSC parent report early childhood group (aged 7 or younger) (*N* = 1511)		
Total	0.78	0.85
Total, item 7 removed	0.76	0.84
AES	0.69	0.71
EOE	0.64	0.66
LST	0.55	0.67
LST, item 7 removed	0.80	0.80
HSC parent report classes of the middle childhood group (aged 8 up to 13) (*N* = 1776)		
Total		0.78
Total, item 7 removed		0.77
AES		0.73
EOE		0.70
LST		0.66
LST, item 7 removed		0.86
HSC parent report adolescent group (aged 14 up to 19) (*N* = 370)		
Total	0.81	0.86
Total, item 7 removed	0.81	0.87
AES	0.66	0.68
EOE	0.78	0.79
LST	0.69	0.77
LST, item 7 removed	0.90	0.90
HSC adolescent self-report (*N* = 250)		
Total	0.67	0.80
AES	0.57	0.63
EOE	0.67	0.69
LST	0.67	0.72

α Cronbach’s alpha, ω McDonald’s omega, *AES * aesthetic sensitivity, *EOE * ease of excitation, *HSC * highly sensitive child scale, LSE low sensory threshold

### Measurement invariance

MI across age groups was assessed using age as a grouping variable, with results presented in Table 3. The CFI for the all-invariance models did not exceed the 0.90 threshold, indicating an unacceptable fit. The RMSEA for the configural model showed a fit that could not be considered acceptable, with values for all models exceeding 0.080. The SRMR and the RNI did not support the acceptability of the model fits. Overall, these results indicate that MI was not supported across age groups.

### Exploratory analysis across age groups, including self- and parent-report: Assessment of global fit, local fit, and internal consistency

An evaluation of the three model variations showed that the bifactorial model provided a better fit for the early childhood group, as shown in Table 2. When calculating the model fit for the other two age groups, a warning was generated during the likelihood ratio test (LRT). The warning suggests that some models that are not typically considered nested based on theoretical structuring may, in fact, not be statistically nested for the purposes of the LRT or that there may have been optimization issues with the less restricted models. Therefore, the models were compared focusing on AIC and BIC, which were lower for the three-factor solution.

When assessing the fit of the bifactorial and three-factor model with and without item 7 across all age groups of the parent report, the CFI was not acceptable for the early childhood-group when item 7 was included but approached acceptability when item 7 was excluded (see Table 3). This was also the case for the two older age-groups, as CFI values did improve with the exclusion of item 7, the adolescent group even reached the cut-offs for acceptability. This pattern was also observed for the RMSEA, where the early-childhood group, even if item 7 was removed, did not show an acceptable fit; this was also the case for the middle childhood group. The adolescent group reached acceptable levels of fit, with and without item 7. The SRMR remained below 1.00 for all age-groups and the RNI was above 0.80, indicating acceptable fit for all groups, both with and without item 7. The RNI for the adolescent report group, even indicated good fit when item 7 was removed.

Reliability, as measured by Cronbach’s alpha and McDonald’s omega, reached acceptable levels for all total scores, with and without item 7, across all age groups of the parent report, with the adolescent group even reaching good Cronbach’s alpha levels, as detailed in Table 4. The AES subscale did marginally reach an acceptable value in the early childhood group, and reached acceptable values in the middle childhood group, but did not reach acceptable values in the adolescent group. For McDonald’s omega concerning the AES subscale only the early childhood group reached satisfactory values, whereas the other two groups did not. For the EOE subscale, only the early childhood age group had Cronbach’s alpha values below the acceptable threshold, whereas the middle childhood and adolescent groups had acceptable values. Considering McDonald’s omega all age groups had satisfactory values in the EOE subscale. For the LST subscale, good reliability was only observed across all age groups when item 7 was removed, with the adolescent groups even achieving excellent Cronbach’s alpha values when item 7 was excluded. For McDonald’s omega all age groups had satisfactory values on the LST subscale.

### Latent profile analysis

**Fig. 1:**
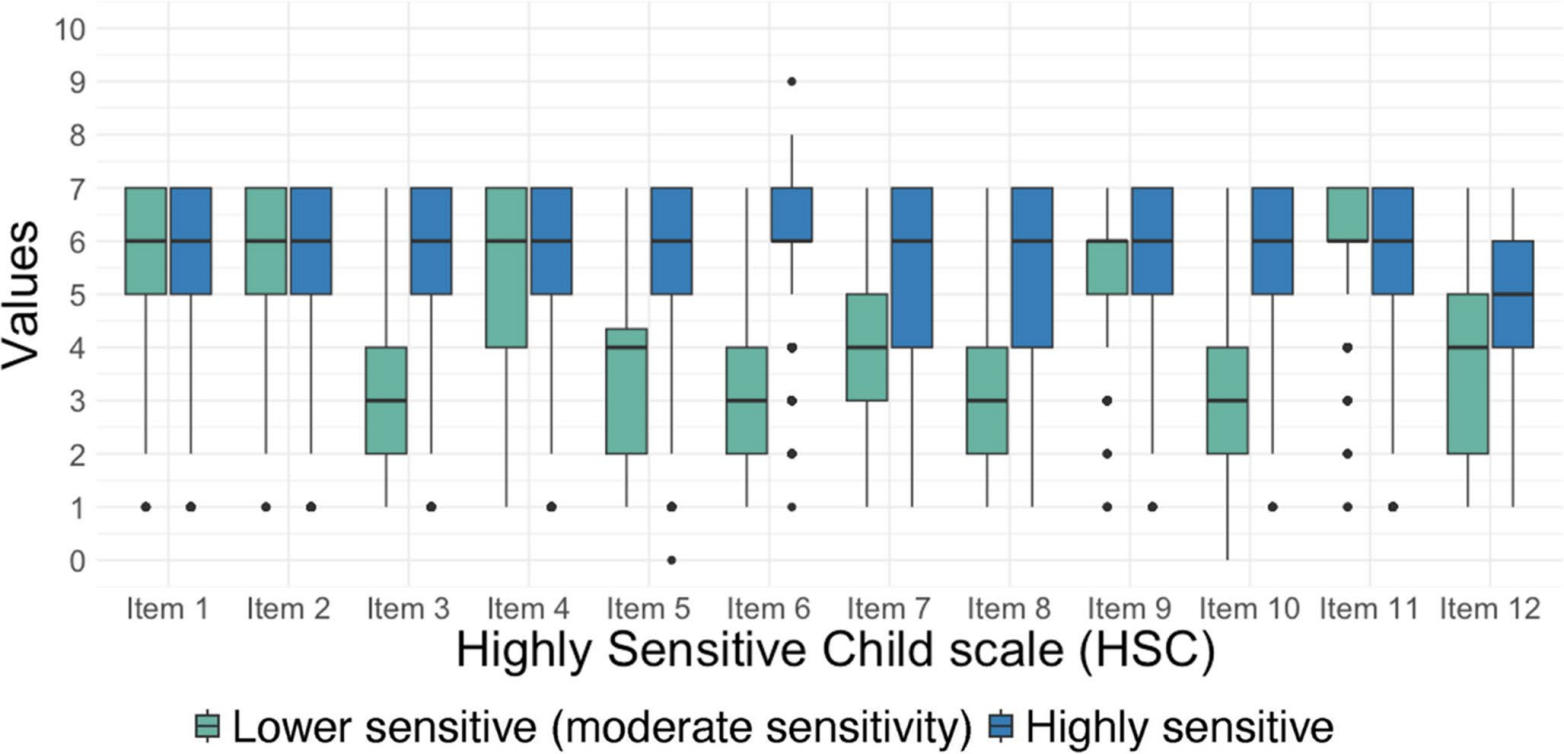
HSC Parent Report Classes Across Age-Groups

The model selected for the total parent report data (Model 3; Pastor et al., 2007) included varying covariances varying variances and consisted of two classes (see Supplementary Table S3): a predominantly low-scoring class, with elevated scores on items 2 and 11 (class 2 [Blue: Highly sensitive]; *n* = 3026, 82.75%, *M* total score = 4.33) and a higher-scoring class, with relatively lower scores on items 2 and 11 (class 1 [Green: Lower sensitive (moderate sensitivity]; *n* = 631, 17.25%, *M* total score = 5.65) (Fig. 1) (see Supplementary Table 8).

**Fig. 2:**
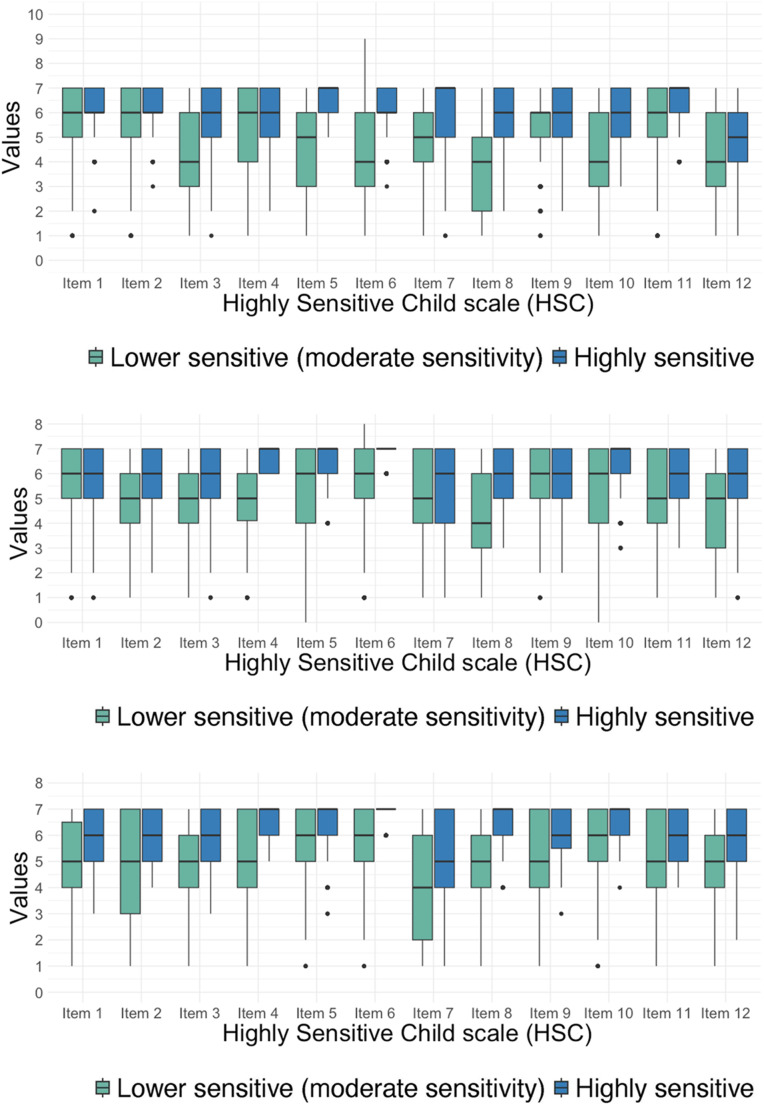
HSC Parent Report Classes by Age Group: Early Childhood (≤ 7 years), Middle Childhood (8–13 years), and Adolescence (14–19 years)

For the early childhood age group, the best fit was a two-profile solution based on a model with varying variances and covariances (see Supplementary Table 4). The classes included an elevated class (class 2 [Blue: Highly sensitive]; *n* = 495, 32.756%, *M* total score = 6.01) and a lower scoring class (class 1 [Green: Lower sensitive (moderate sensitivity]; *n* = 1016, 67.24%, *M* total score = 4.83) (Fig. 2) (see Supplementary Table 9).

**Fig. 3:**
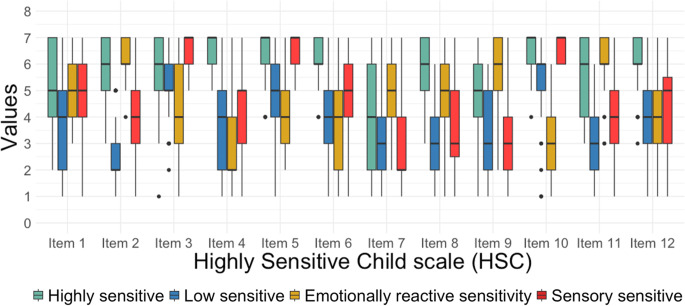
HSC Self-Report Classes of the Adolescent Group

In the middle childhood group, a two-class model with varying covariances and varying variances was chosen (see Supplementary Table 5). The classes consisted of consistently low scores on all HSC items (class 2 [Blue: Highly sensitive]; *n* = 822, 46.28%, *M* total score = 5.11), and consistently high scores across most items (class 1 [Green: Lower sensitive (moderate sensitivity]; *n* = 954, 53.72%, *M* total score = 6.06) (Fig. 3) (see Supplementary Table S10).

For the adolescent parent report group, the best fit was a two-class solution with varying covariances and varying variances (Supplementary Table 6). The classes included a high scoring class (class 2 [Blue: Highly sensitive]; *n* = 203, 54.86%, *M* total score = 6.08) and a low scoring class (class 1 [Green: Lower sensitive (moderate sensitivity]; *n* = 167, 45.14%, *M* total score = 5.11) (see Supplementary Table 11).

In the adolescent self-report version, a four-class solution with zero covariances and varying variances fitted the data best (see Supplementary Table 7). The first class consisted of mostly elevated levels across items (class 1 [Green: Highly Sensitive]; *n* = 49, 19.60%, *M* total score = 5.67), whereas the second and the third class did not consistently differentiate across all items; the second class (class 3 [Yellow: Emotionally reactive sensitive, with moderate to high values on items on e.g., subtle changes, loud noises, and violence]; *n* = 57, 22.80%, *M* total score = 4.60) scored mostly in the mid-range but scored highest on item 2, 7, 9, and 11. The third class (class 4 [Red: Sensory sensitive, with high values on items related to smells, music, and taste]; *n* = 75, 30.00%, *M* total score = 4.61) scored mostly in the middle across items except for items 3,5, and 10 (see Supplementary Table 12). And the fourth class scored mostly low across all items except for item 3 and 10 (class 2 [Blue: Low sensitive]; *n* = 69, 27.60%, *M* total score = 3.90).

## Discussion

The current study investigated the psychometric properties of the German translation of both the self- and parent-reported version of the Highly Sensitive Child Scale (HSC), which captures Environmental Sensitivity in children and adolescents through self- and parent reports (Pluess et al., 2018; Slagt et al., 2018). A growing number of studies have suggested that not only adults but also children and adolescents differ in their ability to register and process external stimuli (Belsky and Pluess 2009b; Pluess 2015; Pluess et al. 2018; Sperati et al. 2022). The current study’s aims were two-fold: First, we aimed to examine the factor structure, including internal consistency and MI across age groups. Second, we aimed to examine the existence of sensitivity groups. Overall, results were in line with our hypotheses, with some exceptions: The analyses showed that the bifactorial model seemed to be the best fit for the parent report and self-report data. However, this was not the case when parents’ reports were stratified by age group. Also, across all age groups, two latent groups were identified. But when looking at specific age groups, only two classes emerged for early, middle childhood, and the adolescent age-group. In contrast, four classes were observed for the adolescent groups (i.e., self-report but not for the parent report data).

### Factor structure, internal consistency, and measurement invariance

In line with our hypothesis, the results revealed that across all age groups, the bifactorial model showed the best fit for both the parent report and the adolescent self-report. The bifactorial model indicates a general sensitivity factor and three subdimensions: Low Sensory Threshold, Ease of Excitation, and Aesthetic Sensitivity. This result is in line with previous studies in children (Pluess et al., 2018; Sperati et al., 2022) and adults (Lionetti et al., 2018; Pluess et al., 2023). However, when the parent report was analyzed separately for each age group, the bifactorial model did not consistently outperform the three-factor model: For the middle childhood and adolescent age groups the three-factor solution fitted better. This might be due to developmental reasons not sufficiently captured in our data.

Similar to previous studies, removing item 7, which captures exposure to violent TV shows, improved model fit for all age groups. In the early childhood group, the improvements in fit indices, particularly CFI and TLI, were most pronounced. This suggests that item 7 may not be a reliable item to capture sensitivity in preschoolers, as younger children, regardless of their sensitivity, are less likely to be exposed to violent TV programs. The present findings support the suggestion of item 7’s replacement with content that reflects interfamilial dynamics (Sperati et al., 2022) or other intense sensory stimuli young children can be exposed to. Although the overall scale showed satisfactory internal consistency, the subscale alphas were low. Previous studies have also reported low internal consistency, especially for the subscales AES and LST (Pluess et al., 2018; Weyn et al., 2019). To address this limitation, Weyn et al. (2021, 2022) used the existing structure of the HCS scale and developed additional items to improve the current 12-item scale. In two different samples, the extended Dutch version showed good internal consistency for the total scale and subscales of the HSC-21. Thus, by including additional items, not only the internal consistency of the subscales could be improved, but it also allows to capture the underlying construct of sensitivity more broadly.

However, in the present findings the low internal consistency of the subscales was less of an issue when considering McDonald’s omega. Excluding item 7 improved the reliability of both translated self-report and parent report concerning the LST subscale in early and middle childhood. In contrast to item 7, the other items on the LST subscale focus on loud noises, which is likely to contribute to a stronger correlation between these items and a weaker link with item 7. This difference may affect the alpha, as the measure is sensitive to item similarity. Nevertheless, the inclusion of diverse, less correlated items can broaden the scope of a scale and enrich its validity (Tavakol & Dennick, 2011), which raises concerns about prematurely discarding such items. This applies especially to the adolescent group, where the removal of item 7 cannot be argued due to reduced age-specific relevance, as discussed in the previous section. Alternative measures such as McDonald’s omega have been proposed, but recent evidence suggests that omega is not necessarily superior to alpha (Deng & Chan, 2016; Malkewitz et al., 2023). In fact, for scales with few items, omega may be less appropriate than alpha (Orcan, 2023). Other researchers argue that for reliability estimates to be meaningful, the underlying validity of the scale must first be established (Borsboom et al., 2004). Therefore, cognitive interviewing, a method that examines how participants understand and respond to questionnaire items, could provide insight into the relevance of item 7. This approach ensures that informed decisions are made regarding the item, thereby increasing the reliability and validity of the scale (Castillo-Díaz & Padilla, 2012).

The MI analysis indicated a lack of MI across age groups. More precisely, configural, metric, and scalar invariance were below the threshold for acceptability. The absence of configural invariance implies that the basic factor structure of the HSC may not hold consistently across age groups, potentially due to age-related differences in how children experience or express sensitivity. The lack of metric and scalar invariance further indicates that group comparisons of factor loadings and item intercepts are not justified, limiting the interpretability of mean differences in total or subscale scores across developmental stages. This is consistent with the finding that the three-factor model provided a better fit than the bifactor model for the middle childhood and adolescent groups. Furthermore, whereas LPA revealed two classes for the early and middle childhood groups, three classes were identified within the adolescent group. These findings suggest that the structural properties of the latent Environmental Sensitivity construct may change across development. As such, caution is warranted when comparing raw HSC scores between young children, older children, and adolescents.

#### Sensitivity groups

The latent profile analyses indicated the existence of three different sensitivity groups when using the parent report: high, medium, and low sensitivity. However, when examining specific age segments within the parent report, two classes emerged for the early, middle, and adolescent age subsamples. These results are somewhat surprising and differ from previous studies with adults (Lionetti et al., 2018) and children and adolescents (Pluess et al., 2018) that have consistently found three sensitivity groups: a low, medium, and high sensitivity group. In the present study, all age groups - except for the adolescents’ self-report sample - showed a lower sensitivity group and a higher sensitivity group, indicating that the groups differ based on their level of sensitivity. Thereby, it is important to note that even though the two groups can be distinguished by their sensitivity level (i.e., lower and higher), the overall scores were still relatively high. Thus, especially compared to previous studies fewer children in the sample were described as low sensitive. The present findings stem from parent report data, which marks a difference from the study by Pluess et al. (2018). In their study, self-report data was used, and on average, children were older than in the present study. It might be that these differences could have affected the results. However, further research, including self-report and parent-report measures, is needed to explore this in more detail. A further unexpected finding was the four-profile solution for the adolescent self-report subsample. Similar to the parent report data, the adolescent self-report data showed a high and low-sensitive group. In addition, two medium-sensitive groups were found. While these two groups show similar overall mean scores, they differed when considering the subscales. While class 3 (Yellow) showed medium scores across the three subscales, group 4 (Red) showed a lot of variability across the different subscales, scoring high on the AES subscale, medium on the EOE subscale, and low on the LST subscale. To sum up, the present findings suggest that individual sensitivity becomes more nuanced over time, with less variability in early and middle childhood and great variability in adolescence. One reason why this may not have emerged in the adolescent parent report is that it becomes increasingly challenging for parents to accurately report on their adolescent’s personality development during this development phase (Göllner et al., 2016).

#### Strengths and limitations

This study has several strengths: It is the first study to explore the psychometric properties of the German translation of both the HSC and the HSC-PR. Also, a large data set with data from Switzerland, Germany, and Austria was analyzed. Lastly, the analyses were carried out in accordance with previously published studies for other language versions (Dong et al., 2022; Liu et al., 2023; Pluess et al., 2018; Weyn et al., 2021, 2022). Despite these strengths, our study has some limitations: First, we used different data sets collected in varying contexts, and hence included a heterogeneous set of participants. Second, item 7 (violent TV programs) showed limited reliability particularly in younger children, likely due to age-related differences in exposure. This highlights the need to revise or replace this item for early childhood samples. Third, the cross-sectional nature of the datasets used precluded the calculation of test-retest reliability and predictive validity. This limitation restricts our ability to assess the retest-stability of the measures over time and their predictive validity. Fourth, we did not assess construct validity, an aspect that has been explored in other validation studies. Such studies have included various related constructs such as personality (Weyn et al., 2019), temperament, parenting styles, and psychological functioning (Weyn et al. 2021, 2022) to establish construct validity. Lastly, the sample size between the self-report and parent-report versions of the HSC varies substantially restricting the comparison of psychometric properties across the two scales. Addressing these issues in future research would enhance our understanding of the validity of the measures in different contexts and their relationship with related constructs.

#### Practical implications

Despite the listed psychometric shortcomings, the German versions of the HSC and HSC-PR demonstrates sufficient validity for practical applications in its current form. From a developmental perspective, these scales enable researchers to examine how sensitivity may interact with environmental influences such as parenting or educational settings (e.g., preschool, school) across different developmental stages. This, in turn could enhance our understanding of how sensitivity-environment interactions unfold over time (Belsky and Pluess 2009a). From an applied perspective, the scales may serve as a screening tool, to identify children who could benefit from environmental modifications especially in emotionally or sensorily demanding contexts. Nonetheless, results should be interpreted cautiously across age groups and subscales, given the limitations in MI and subscale reliability. Researchers and practitioners are encouraged to consider these issues when using or adapting the HSC in German-speaking populations.

## Conclusion and future directions

The present study has investigated the psychometric properties of the HSC and HSC-PR in a large sample from three different German speaking countries. The bifactorial model, with one general sensitivity factor and three subdimensions (i.e., EOE, LST and AES) fits our data best. The study provides valuable insights into the psychometric characteristics of the scale and contributes to a better understanding of how the construct of sensitivity manifests across development, diverse populations and age groups. Future research should explore age-appropriateness of all items, specifically item 7, and assess construct validity of the German version.

## Supplementary information

Below is the link to the electronic supplementary material.

Supplementary Material 1 (DOCX 107 KB)

## Data Availability

Data available on request.
