# Supplementary material for: The German version of the highly sensitive child scale: Psychometric properties and identification of sensitivity groups
Source: Curr Psychol. 2026 Mar 23;45(7):738. doi: 10.1007/s12144-026-09244-w (PMC13009142; doi:10.1007/s12144-026-09244-w)
Supplement: Supplementary file 1 — Supplementary Material 1 (DOCX 107 KB) [file 12144_2026_9244_MOESM1_ESM.docx]

**Supplementary Data**

# Table S1

# *Factor loadings of bifactorial model after CFA for HSC parent report (N = 2988)*

|  | |  | |  | |  | |  | |  | | 95% CI | | |
| --- | --- | --- | --- | --- | --- | --- | --- | --- | --- | --- | --- | --- | --- | --- |
| Latent factor | Indicator | | *B* | | *SE* | | *z* | | *p* | | β | | *LL* | *UL* |
|  |  | |  | |  | |  | |  | |  | |  |  |
| AES | 1 | | -0.007 | | 0.027 | | -0.269 | | 0.788 | | -0.006 | | -0.059 | 0.045 |
|  | 3 | | 0.064 | | 0.043 | | 1.490 | | 0.136 | | 0.042 | | -0.020 | 0.149 |
|  | 5 | | 1.196 | | 0.026 | | 45.287 | | 0.000 | | 0.745 | | 1.144 | 1.248 |
|  | 10 | | 0.123 | | 0.043 | | 2.869 | | 0.004 | | 0.079 | | 0.039 | 0.207 |
|  |  | |  | |  | |  | |  | |  | |  |  |
|  |  | |  | |  | |  | |  | |  | |  |  |
| EOE | 4 | | 0.177 | | 0.031 | | 5.682 | | 0.000 | | 0.128 | | 0.116 | 0.238 |
|  | 6 | | 0.043 | | 0.037 | | 1.148 | | 0.251 | | 0.026 | | -0.030 | 0.115 |
|  | 8 | | 0.139 | | 0.041 | | 3.424 | | 0.001 | | 0.077 | | 0.059 | 0.218 |
|  | 9 | | 0.146 | | 0.025 | | 5.742 | | 0.000 | | 0.114 | | 0.096 | 0.195 |
|  | 12 | | 1.434 | | 0.023 | | 62.708 | | 0.000 | | 0.863 | | 1.389 | 1.478 |
|  |  | |  | |  | |  | |  | |  | |  |  |
|  |  | |  | |  | |  | |  | |  | |  |  |
| LST | 2 | | 1.107 | | 0.053 | | 20.932 | | 0.000 | | 0.779 | | 1.004 | 1.211 |
|  | 7 | | 0.307 | | 0.036 | | 8.532 | | 0.000 | | 0.172 | | 0.236 | 0.377 |
|  | 11 | | 1.379 | | 0.063 | | 21.885 | | 0.000 | | 0.936 | | 1.256 | 1.503 |
|  |  | |  | |  | |  | |  | |  | |  |  |

*Notes.* *B* = unstandardized loadings. *SE* = standard error. β = standardized loadings. *z* = z-value*. p* = p-value. CI = confidence interval. LL = lower limit. UL = upper limit. AES = Aesthetic Sensitivity. EOE = Ease of Excitation. LST = Low Sensory Threshold. HSC = Highly Sensitive Child Scale.

#

# Table S2

*Factor Loadings of Bifactorial Model After CFA for HSC Adolescent Self-Report (N = 231)*

|  | |  | |  | |  | | |  | |  | | 95% CI | |
| --- | --- | --- | --- | --- | --- | --- | --- | --- | --- | --- | --- | --- | --- | --- |
| Latent factor | Indicator | | *B* | | *SE* | | *z* | *p* | | β | | *LL* | | *UL* |
|  |  | |  | |  | |  |  | |  | |  | |  |
| AES | 1 | | 0.327 | | 0.121 | | 2.712 | 0.007 | | 0.204 | | 0.091 | | 0.564 |
|  | 3 | | 0.878 | | 0.130 | | 6.750 | 0.000 | | 0.583 | | 0.623 | | 1.132 |
|  | 5 | | 0.864 | | 0.105 | | 8.202 | 0.000 | | 0.603 | | 0.657 | | 1.070 |
|  | 10 | | 1.093 | | 0.151 | | 7.233 | 0.000 | | 0.640 | | 0.797 | | 1.389 |
|  |  | |  | |  | |  |  | |  | |  | |  |
|  |  | |  | |  | |  |  | |  | |  | |  |
| EOE | 4 | | 1.206 | | 0.128 | | 9.408 | 0.000 | | 0.640 | | 0.955 | | 1.457 |
|  | 6 | | 1.315 | | 0.102 | | 12.927 | 0.000 | | 0.780 | | 1.116 | | 1.515 |
|  | 8 | | 0.827 | | 0.116 | | 7.105 | 0.000 | | 0.484 | | 0.599 | | 1.055 |
|  | 9 | | -0.041 | | 0.126 | | -0.322 | 0.747 | | -0.022 | | -0.288 | | 0.207 |
|  | 12 | | 0.866 | | 0.132 | | 6.581 | 0.000 | | 0.498 | | 0.608 | | 1.123 |
|  |  | |  | |  | |  |  | |  | |  | |  |
|  |  | |  | |  | |  |  | |  | |  | |  |
| LST | 2 | | 0.584 | | 0.254 | | 2.298 | 0.022 | | 0.307 | | 0.086 | | 1.083 |
|  | 7 | | -0.553 | | 0.454 | | -1.219 | 0.223 | | -0.293 | | -1.442 | | 0.336 |
|  | 11 | | 0.592 | | 0.270 | | 2.190 | 0.028 | | 0.336 | | 0.062 | | 1.121 |
|  |  | |  | |  | |  |  | |  | |  | |  |

*Notes.* *B* = unstandardized loadings. *SE* = standard error. β = standardized loadings. *z* = z-value*. p* = p-value. CI = confidence interval. LL = lower limit. UL = upper limit. AES = Aesthetic Sensitivity. EOE = Ease of Excitation. LST = Low Sensory Threshold. HSC = Highly Sensitive Child Scale.

# Table S3

# *Model Fit Indices for LPA Model Comparison, HSC Parent Report Total Sample*

| Model | *Log Likelihood* | AIC | BIC | CAIC | | aBIC | ICL | Entropy | *p* (BLRT) |
| --- | --- | --- | --- | --- | --- | --- | --- | --- | --- |
| Zero covariances – equal variances | | | | | | | | | |
| 1-Class | -62262.70 | 124573.4 | 124722.3 | 124746.3 | | 124646.0 | -124722.3 | 1.000 | – |
| 2-Class | -58285.97 | 116645.9 | 116875.5 | 116912.5 | | 116757.9 | -117047.9 | 0.931 | < 0.01 |
| 3-Class | -56600.38 | 113300.8 | 113611.0 | 113661.0 | | 113452.1 | -113991.8 | 0.904 | < 0.01 |
| 4-Class | -56113.53 | 112353.1 | 112743.9 | 112806.9 | | 112543.8 | -113715.3 | 0.813 | < 0.01 |
| 5-Class | -55541.44 | 111234.9 | 111706.4 | 111782.4 | | 111464.9 | -112694.2 | 0.832 | < 0.01 |
| 6-Class | -55403.23 | 110984.5 | 111536.6 | 111625.6 | | 111253.8 | -112853.3 | 0.7976 | < 0.01 |
| Zero covariances – varying variances | | | | | | | | | |
| 1-Class | -62262.70 | 124573.4 | 124722.3 | 124746.3 | | 124646.0 | -124722.3 | 1.000 | – |
| 2-Class | -57141.95 | 114381.9 | 114685.9 | 114734.9 | | 114530.2 | -115060.2 | 0.849 | < 0.01 |
| Equal covariances – equal variances | | | | |  | | | | |
| 1-Class | -55661.63 | 111503.3 | 112061.7 | 112151.7 | | 111775.7 | -112061.7 | 1.000 | – |
| 2-Class | -54865.42 | 109936.8 | 110575.9 | 110678.9 | | 110248.6 | -110739.9 | 0.932 | < 0.01 |
| 3-Class | -54865.33 | 109962.7 | 110682.4 | 110798.4 | | 110313.8 | -112488.5 | 0.571 | < 0.01 |
| 4-Class | -54468.43 | 109194.9 | 109995.2 | 110124.2 | | 109585.3 | -112162.2 | 0.609 | < 0.01 |
| 5-Class | -54173.55 | 108631.1 | 109512.1 | 109654.1 | | 109060.9 | -110510.6 | 0.827 | < 0.01 |
| 6-Class | -54362.07 | 109034.1 | 109995.8 | 110150.8 | | 109503.3 | -111518.4 | 0.725 | < 0.01 |
| Varying covariances – varying variances | | | | | | | | | |
| 1-Class | -55661.63 | 111503.3 | 112061.7 | 112151.7 | | 111775.7 | -112061.7 | 1.000 | – |
| **2-Class** | **-53371.42** | **107104.8** | **108227.8** | **108408.8** | | **107652.7** | **-108854.4** | **0.753** | **< 0.01** |
| Equal covariances – varying variances | | | | | | | | | |
| Model did not converge | | | | | | | | | |
| Varying covariances – equal variances | | | | | | | | | |
| Model did not converge | | | | | | | | | |

*Notes.* AIC = Akaike Information Criterion. BIC = Bayesian Information Criterion. aBIC = adjusted Bayesian Information Criterion. CAIC = Consistent Aikake Information Criterion. ICL = Integrated completed likelihood. BLRT = Bootstrapped Likelihood Ratio Test. *p* = p-value.

#

# Table S4

*Model Fit Indices for LPA Model Comparison, HSC Parent Report Early Childhood Group (Aged 7 or Younger)*

| Model | *Log Likelihood* | AIC | BIC | CAIC | | aBIC | ICL | Entropy | *p* (BLRT) |
| --- | --- | --- | --- | --- | --- | --- | --- | --- | --- |
| Zero covariances – equal variances | | | | | | | | | |
| 1-Class | -25722.19 | 51492.38 | 51620.08 | 51644.08 | | 51543.83 | -51620.08 | 1.000 | – |
| 2-Class | -23906.83 | 47887.66 | 48084.52 | 48121.52 | | 47966.98 | -48188.14 | 0.901 | < 0.01 |
| 3-Class | -23331.21 | 46762.42 | 47028.45 | 47078.45 | | 46869.61 | -47187.08 | 0.902 | < 0.01 |
| 4-Class | -23097.92 | 46321.84 | 46657.03 | 46720.03 | | 46456.90 | -47041.70 | 0.821 | < 0.01 |
| 5-Class | -22968.91 | 46089.82 | 46494.18 | 46570.18 | | 46252.75 | -47019.87 | 0.788 | < 0.01 |
| 6-Class | -22839.50 | 45857.00 | 46330.53 | 46419.53 | | 46047.80 | -46863.16 | 0.805 | < 0.01 |
| Zero covariances – varying variances | | | | | | | | | |
| 1-Class | -25722.19 | 51492.38 | 51620.08 | 51644.08 | | 51543.83 | -51620.08 | 1.000 | – |
| 2-Class | -23733.36 | 47564.72 | 47825.42 | 47874.42 | | 47669.76 | -47960.07 | 0.872 | < 0.01 |
| Equal covariances – equal variances | | | | |  | | | | |
| 1-Class | -22923.33 | 46026.67 | 46505.51 | 46595.51 | | 46219.61 | -46505.51 | 1.000 | – |
| 2-Class | -22721.23 | 45648.45 | 46196.47 | 46299.47 | | 45869.26 | -46333.68 | 0.863 | < 0.01 |
| 3-Class | -22526.78 | 45285.57 | 45902.75 | 46018.75 | | 45534.25 | -46119.87 | 0.860 | < 0.01 |
| 4-Class | -22478.63 | 45215.26 | 45901.61 | 46030.61 | | 45491.81 | -46215.71 | 0.842 | < 0.01 |
| 5-Class | -22439.37 | 45162.75 | 45918.26 | 46060.26 | | 45467.16 | -46239.29 | 0.857 | < 0.01 |
| 6-Class | -22353.44 | 45016.88 | 45841.56 | 45996.56 | | 45349.16 | -46265.47 | 0.835 | < 0.01 |
| Varying covariances – varying variances | | | | | | | | | |
| 1-Class | -22923.33 | 46026.67 | 46505.51 | 46595.51 | | 46219.61 | -46505.51 | 1.000 | **–** |
| **2-Class** | **-22125.09** | **44612.17** | **45575.19** | **45756.19** | | **45000.20** | **-45739.42** | **0.835** | **< 0.01** |
| 3-Class | -21636.10 | 43816.20 | 45263.38 | 45535.38 | | 44399.31 | -45613.28 | 0.770 | < 0.01 |
| Equal covariances – varying variances | | | | | | | | | |
| Model did not converge | | | | | | | | | |
| Varying covariances – equal variances | | | | | | | | | |
| Model did not converge | | | | | | | | | |

*Notes.* AIC = Akaike Information Criterion. BIC = Bayesian Information Criterion. aBIC = adjusted Bayesian Information Criterion. CAIC = Consistent Aikake Information Criterion. ICL = Integrated completed likelihood. BLRT = Bootstrapped Likelihood Ratio Test. *p* = p-value.

#

# Table S5

# *Model Fit Indices for LPA Model Comparison, HSC Parent-Report Classes of the Middle Childhood Group (Aged 8 up to 13)*

| Model | *Log Likelihood* | AIC | BIC | CAIC | | aBIC | ICL | Entropy | *p* (BLRT) |
| --- | --- | --- | --- | --- | --- | --- | --- | --- | --- |
| Zero covariances – equal variances | | | | | | | | | |
| 1-Class | -30234.42 | 60516.83 | 60648.40 | 60672.40 | | 60572.16 | -60648.40 | 1.000 | – |
| 2-Class | -28707.54 | 57489.08 | 57691.92 | 57728.92 | | 57574.37 | -57770.87 | 0.932 | < 0.01 |
| 3-Class | -27832.96 | 55765.92 | 56040.02 | 56090.02 | | 55881.18 | -56242.23 | 0.895 | < 0.01 |
| 4-Class | -27520.43 | 55166.85 | 55512.23 | 55575.23 | | 55312.08 | -55809.98 | 0.876 | < 0.01 |
| 5-Class | -27296.63 | 54745.26 | 55161.90 | 55237.90 | | 54920.46 | -55582.55 | 0.849 | < 0.01 |
| 6-Class | -27163.28 | 54504.55 | 54992.46 | 55081.46 | | 54709.72 | -55606.79 | 0.804 | < 0.01 |
| Zero covariances – varying variances | | | | | | | | | |
| 1-Class | -30234.42 | 60516.83 | 60648.40 | 60672.40 | | 60572.16 | -60648.40 | 1.000 | – |
| 2-Class | -27828.65 | 55755.31 | 56023.93 | 56072.93 | | 55868.26 | -56217.30 | 0.836 | < 0.01 |
| Equal covariances – equal variances | | | | |  | | | | |
| 1-Class | -27275.98 | 54731.95 | 55225.34 | 55315.34 | | 54939.42 | -55225.34 | 1.000 | – |
| 2-Class | -26840.59 | 53887.18 | 54451.84 | 54554.84 | | 54124.61 | -54484.79 | 0.973 | < 0.01 |
| 3-Class | -26840.81 | 53913.63 | 54549.55 | 54665.55 | | 54181.03 | -55610.84 | 0.509 | 0.891 |
| 4-Class | -26682.86 | 53623.72 | 54330.91 | 54459.91 | | 53921.09 | -55479.14 | 0.580 | < 0.01 |
| 5-Class | -26626.24 | 53536.48 | 54314.94 | 54456.94 | | 53863.82 | -55963.92 | 0.574 | < 0.01 |
| 6-Class | -26626.17 | 53562.33 | 54412.06 | 54567.06 | | 53919.64 | -56043.69 | 0.559 | 0.109 |
| Varying covariances – varying variances | | | | | | | | | |
| 1-Class | -27275.98 | 54731.95 | 55225.34 | 55315.34 | | 54939.42 | -55225.34 | 1.000 | **–** |
| **2-Class** | **-26044.08** | **52450.16** | **53442.43** | **53623.43** | | **52867.40** | **-53677.55** | **0.792** | **< 0.01** |
| Equal covariances – varying variances | | | | | | | | | |
| Model did not converge | | | | | | | | | |
| Varying covariances – equal variances | | | | | | | | | |
| Model did not converge | | | | | | | | | |

*Notes.* AIC = Akaike Information Criterion. BIC = Bayesian Information Criterion. aBIC = adjusted Bayesian Information Criterion. CAIC = Consistent Aikake Information Criterion. ICL = Integrated completed likelihood. BLRT = Bootstrapped Likelihood Ratio Test. *p* = p-value.

# Table S6

# *Model Fit Indices for LPA Model Comparison, HSC Parent Report Adolescent Group (Aged 14 up to 19)*

| Model | *Log Likelihood* | AIC | BIC | CAIC | | aBIC | ICL | Entropy | *p* (BLRT) |
| --- | --- | --- | --- | --- | --- | --- | --- | --- | --- |
| Zero covariances – equal variances | | | | | | | | | |
| 1-Class | -6294.079 | 12636.16 | 12730.08 | 12754.08 | | 12653.94 | -12730.08 | 1.000 | – |
| 2-Class | -5928.917 | 11931.83 | 12076.63 | 12113.63 | | 11959.25 | -12093.42 | 0.929 | < 0.01 |
| 3-Class | -5850.975 | 11801.95 | 11997.63 | 12047.63 | | 11838.99 | -12053.28 | 0.851 | < 0.01 |
| 4-Class | -5682.283 | 11490.57 | 11737.12 | 11800.12 | | 11537.24 | -11793.02 | 0.882 | < 0.01 |
| 5-Class | -5615.634 | 11383.27 | 11680.69 | 11756.69 | | 11439.57 | -11731.24 | 0.900 | < 0.01 |
| 6-Class | -5591.477 | 11360.95 | 11709.26 | 11798.26 | | 11426.89 | -11781.97 | 0.882 | < 0.01 |
| Zero covariances – varying variances | | | | | | | | | |
| 1-Class | -6294.079 | 12636.16 | 12730.08 | 12754.08 | | 12653.94 | -12730.08 | 1.000 | – |
| Equal covariances – equal variances | | | | |  | | | | |
| 1-Class | -5557.471 | 11294.94 | 11647.16 | 11737.16 | | 11361.62 | -11647.16 | 1.000 | – |
| 2-Class | -5523.042 | 11252.08 | 11655.17 | 11758.17 | | 11328.39 | -11689.69 | 0.841 | < 0.01 |
| 3-Class | -5448.700 | 11129.40 | 11583.37 | 11699.37 | | 11215.34 | -11609.84 | 0.928 | < 0.01 |
| 4-Class | -5475.053 | 11208.11 | 11712.95 | 11841.95 | | 11303.67 | -11835.89 | 0.745 | 0.871 |
| 5-Class | -5474.736 | 11233.47 | 11789.19 | 11931.19 | | 11338.67 | -11979.06 | 0.652 | 1.000 |
| 6-Class | -5403.525 | 11117.05 | 11723.64 | 11878.64 | | 11231.88 | -11830.00 | 0.818 | < 0.01 |
| 7-Class | -5376.556 | 11089.11 | 11746.58 | 11914.58 | | 11213.57 | -11836.79 | 0.859 | < 0.01 |
| Varying covariances – varying variances | | | | | | | | | |
| 1-Class | -5557.471 | 11294.94 | 11647.16 | 11737.16 | | 11361.62 | -11647.16 | 1.000 | **–** |
| **2-Class** | **-5209.095** | **10780.19** | **11488.53** | **11669.53** | | **10914.28** | **-11530.11** | **0.817** | **< 0.01** |
| Equal covariances – varying variances | | | | | | | | | |
| Model did not converge | | | | | | | | | |
| Varying covariances – equal variances | | | | | | | | | |
| Model did not converge | | | | | | | | | |

*Notes.* AIC = Akaike Information Criterion. BIC = Bayesian Information Criterion. aBIC = adjusted Bayesian Information Criterion. CAIC = Consistent Aikake Information Criterion. ICL = Integrated completed likelihood. BLRT = Bootstrapped Likelihood Ratio Test. *p* = p-value.

#

# Table S7

# *Model Fit Indices for LPA Model Comparison, HSC Self-Report Classes of the Adolescent Group*

| Model | *Log Likelihood* | AIC | BIC | CAIC | | aBIC | ICL | Entropy | *p* (BLRT) |
| --- | --- | --- | --- | --- | --- | --- | --- | --- | --- |
| Zero covariances – equal variances | | | | | | | | | |
| 1-Class | -4250.804 | 8549.607 | 8634.122 | 8658.122 | | 8558.040 | -8634.122 | 1.000 | – |
| 2-Class | -4029.963 | 8133.926 | 8264.220 | 8301.220 | | 8146.927 | -8273.368 | 0.942 | < 0.01 |
| 3-Class | -3854.536 | 7809.072 | 7985.145 | 8035.145 | | 7826.641 | -8005.997 | 0.909 | < 0.01 |
| 4-Class | -3792.751 | 7711.502 | 7933.354 | 7996.354 | | 7733.638 | -7961.083 | 0.906 | < 0.01 |
| 5-Class | -3770.507 | 7693.014 | 7960.645 | 8036.645 | | 7719.719 | -8001.644 | 0.893 | < 0.05 |
| 6-Class | -3728.820 | 7635.639 | 7949.049 | 8038.049 | | 7666.912 | -7997.160 | 0.882 | < 0.01 |
| Zero covariances – varying variances | | | | | | | | | |
| 1-Class | -4250.804 | 8549.607 | 8634.122 | 8658.122 | | 8558.040 | -8634.122 | 1.000 | – |
| 2-Class | -3969.409 | 8036.817 | 8209.369 | 8258.369 | | 8054.035 | -8217.983 | 0.951 | < 0.01 |
| 3-Class | -3777.867 | 7703.734 | 7964.322 | 8038.322 | | 7729.736 | -7976.072 | 0.950 | < 0.01 |
| **4-Class** | **-3677.946** | **7553.893** | **7902.518** | **8001.518** | | **7588.679** | **-7934.935** | **0.896** | **< 0.01** |
| Equal covariances – equal variances | | | | |  | | | | |
| 1-Class | -3775.196 | 7730.393 | 8047.324 | 8137.324 | | 7762.017 | -8047.324 | 1.000 | – |
| 2-Class | -3707.799 | 7621.598 | 7984.308 | 8087.308 | | 7657.789 | -7988.941 | 0.962 | < 0.01 |
| 3-Class | -3693.182 | 7618.364 | 8026.854 | 8142.854 | | 7659.124 | -8064.336 | 0.852 | < 0.01 |
| 4-Class | -3668.820 | 7595.640 | 8049.909 | 8178.909 | | 7640.968 | -8088.508 | 0.869 | 0.822 |
| 5-Class | -3654.452 | 7592.905 | 8092.952 | 8234.952 | | 7642.800 | -8141.986 | 0.863 | 1.000 |
| 6-Class | -3631.863 | 7573.726 | 8119.552 | 8274.552 | | 7628.189 | -8172.700 | 0.852 | < 0.01 |
| Varying covariances – varying variances | | | | | | | | | |
| 1-Class | -3775.196 | 7730.393 | 8047.324 | 8137.324 | | 7762.017 | -8047.324 | 1.000 | **–** |
| 2-Class | -3572.090 | 7506.181 | 8143.565 | 8324.565 | | 7569.780 | -8146.752 | 0.980 | < 0.01 |
| 3-Class | -3467.280 | 7478.560 | 8436.398 | 8708.398 | | 7574.134 | -8444.641 | 0.960 | < 0.05 |
| 4-Class | -3360.316 | 7446.633 | 8724.923 | 9087.923 | | 7574.182 | -8737.793 | 0.949 | < 0.01 |
| 5-Class | -3252.597 | 7413.195 | 9011.938 | 9465.938 | | 7572.719 | -9020.891 | 0.969 | 0.109 |
| 6-Class | -3125.353 | 7340.706 | 9259.902 | 9804.902 | | 7532.206 | -9264.720 | 0.983 | 0.020 |
| Equal covariances – varying variances | | | | | | | | | |
| Model did not converge | | | | | | | | | |
| Varying covariances – equal variances | | | | | | | | | |
| Model did not converge | | | | | | | | | |

*Notes.* AIC = Akaike Information Criterion. BIC = Bayesian Information Criterion. aBIC = adjusted Bayesian Information Criterion. CAIC = Consistent Aikake Information Criterion. ICL = Integrated completed likelihood. BLRT = Bootstrapped Likelihood Ratio Test. *p* = p-value.

# Table S8

# *Descriptive Statistics of Classes of the HSC Parent Report Total Sample*

|  | | **Green:**  **Lower sensitive**  **(moderate sensitivity)**  *n* = 631 | **Blue:**  **Highly sensitive**  *n* = 3026 |
| --- | --- | --- | --- |
| Age, *M* (*range)* | | 6.2 (1.7) | 9.0 (3.5) |
| Gender, *n* (%) | |  |  |
| Male | | 289 (46%) | 1,539 (51%) |
| Female | | 342 (54%) | 1,473 (49%) |
| Prefer not to say | | 0 (0%) | 14 (0.5%) |
| HSC | | 5.57 (1.31) | 5.73 (1.29) |
| Item 1 | 5.89 (1.16) | 5.57 (1.45) |  |
| Item 2 | 3.39 (1.45) | 5.47 (1.31) |  |
| Item 3 | 5.24 (1.65) | 5.87 (1.29) |  |
| Item 4 | 3.51 (1.58) | 6.02 (1.22) |  |
| Item 5 | 2.82 (1.17) | 6.16 (1.03) |  |
| Item 6 | 4.12 (1.49) | 5.36 (1.76) |  |
| Item 7 | 2.77 (1.37) | 5.36 (1.54) |  |
| Item 8 | 5.68 (1.13) | 5.68 (1.31) |  |
| Item 9 | 3.34 (1.41) | 5.91 (1.18) |  |
| Item 10 | 6.14 (1.12) | 5.59 (1.50) |  |
| Item 11 | 3.51 (1.62) | 5.08 (1.53) |  |
| Item 12 | 15.8 (3.5) | 23.1 (3.2) |  |
| Total sum core | 68 (8) | 52 (8) |  |
| Total mean core | 5.65 (0.70) | 4.33 (0.66) |  |
| AES sum score | 23.1 (3.2) | 15.8 (3.5) |  |
| EOE sum score | 28.1 (4.5) | 20.0 (4.0) |  |
| LST sum score | 16.5 (3.6) | 16.1 (2.6) |  |
| AES mean score | 5.78 (0.81) | 3.95 (0.86) |  |
| EOE mean score | 5.63 (0.90) | 4.01 (0.80) |  |
| LST mean score | 5.51 (1.21) | 5.38 (0.86) |  |

*Notes.* HSC = Highly Sensitive Child Scale. *M* = mean. *N* = sample size. *SD* = standard

deviation.

# Table S9

# *Descriptive Statistics of Classes of the HSC Parent Report Early Childhood Group (Aged 7 or Younger)*

|  | | **Green:**  **Lower sensitive**  **(moderate sensitivity)**  *n* = 1016 | **Blue:**  **Highly sensitive**  *n* = 495 |
| --- | --- | --- | --- |
| Age, *M* (*range)* | | 5.49 (1.08) | 5.55 (1.30) |
| Gender, *n* (%) | |  |  |
| Male | | 509 (50%) | 235 (47%) |
| Female | | 505 (50%) | 259 (52%) |
| Prefer not to say | | 2 (0.2%) | 1 (0.2%) |
| HSC | | 5.70 (1.31) | 6.14 (1.02) |
| Item 1 | 5.55 (1.37) | 6.27 (0.86) |  |
| Item 2 | 4.31 (1.66) | 5.67 (1.24) |  |
| Item 3 | 5.25 (1.59) | 5.85 (1.10) |  |
| Item 4 | 4.58 (1.76) | 6.65 (0.48) |  |
| Item 5 | 4.32 (1.83) | 6.23 (0.88) |  |
| Item 6 | 4.76 (1.69) | 5.97 (1.40) |  |
| Item 7 | 3.73 (1.81) | 5.91 (1.12) |  |
| Item 8 | 5.47 (1.28) | 5.88 (1.15) |  |
| Item 9 | 4.41 (1.70) | 6.12 (1.02) |  |
| Item 10 | 5.71 (1.47) | 6.31 (0.89) |  |
| Item 11 | 4.18 (1.71) | 5.10 (1.44) |  |
| Item 12 | 19.0 (4.3) | 24.6 (2.6) |  |
| Total sum core | 58 (9) | 72 (6) |  |
| Total mean core | 4.83 (0.77) | 6.01 (0.50) |  |
| AES sum score | 19.0 (4.3) | 24.6 (2.6) |  |
| EOE sum score | 23.0 (5.0) | 29.0 (3.6) |  |
| LST sum score | 16.0 (3.1) | 18.6 (2.3) |  |
| AES mean score | 4.75 (1.09) | 6.15 (0.66) |  |
| EOE mean score | 4.59 (0.99) | 5.79 (0.72) |  |
| LST mean score | 5.34 (1.02) | 6.18 (0.76) |  |

*Notes.* HSC = Highly Sensitive Child Scale. *M* = mean. *N* = sample size. *SD* = standard

deviation.

# Table S10

# *Descriptive Statistics of Classes of the HSC Parent-Report Classes of the Middle Childhood Group (Aged 8 up to 13)*

|  | | **Green:**  **Lower sensitive**  **(moderate sensitivity)**  *n* = 954 | **Blue:**  **Highly sensitive**  *n* = 822 |
| --- | --- | --- | --- |
| Age, *M* (*range)* | | 9.30 (1.71) | 9.94 (1.68) |
| Gender, *n* (%) | |  |  |
| Male | | 498 (52%) | 430 (52%) |
| Female | | 451 (47%) | 386 (47%) |
| Prefer not to say | | 5 (0.5%) | 6 (0.7%) |
| HSC | | 5.59 (1.29) | 5.69 (1.34) |
| Item 1 | 5.12 (1.63) | 5.96 (1.10) |  |
| Item 2 | 5.01 (1.50) | 5.70 (1.23) |  |
| Item 3 | 5.29 (1.36) | 6.73 (0.44) |  |
| Item 4 | 5.28 (1.67) | 6.40 (0.80) |  |
| Item 5 | 5.30 (1.54) | 6.79 (0.40) |  |
| Item 6 | 4.95 (1.81) | 5.62 (1.67) |  |
| Item 7 | 4.39 (1.74) | 6.04 (1.01) |  |
| Item 8 | 5.47 (1.36) | 6.05 (1.07) |  |
| Item 9 | 5.43 (1.52) | 6.18 (1.00) |  |
| Item 10 | 5.05 (1.70) | 6.04 (1.04) |  |
| Item 11 | 4.43 (1.64) | 5.58 (1.30) |  |
| Item 12 | 21.3 (4.1) | 24.0 (2.9) |  |
| Total sum core | 61 (9) | 73 (6) |  |
| Total mean core | 5.11 (0.74) | 6.06 (0.46) |  |
| AES sum score | 21.3 (4.1) | 24.0 (2.9) |  |
| EOE sum score | 24.9 (4.7) | 31.2 (2.5) |  |
| LST sum score | 15.1 (3.8) | 17.6 (2.9) |  |
| AES mean score | 5.33 (1.03) | 5.99 (0.71) |  |
| EOE mean score | 4.98 (0.95) | 6.24 (0.50) |  |
| LST mean score | 5.04 (1.28) | 5.87 (0.96) |  |

*Notes.* HSC = Highly Sensitive Child Scale. *M* = mean. *N* = sample size. *SD* = standard

deviation.

# Table S11

# *Descriptive Statistics of Classes of the HSC Parent-Report Classes of the Adolescent Group (Aged 14 up to 19)*

|  | | **Green:**  **Lower sensitive**  **(moderate sensitivity)**  *n* = 167 | **Blue:**  **Highly sensitive**  *n* = 203 |
| --- | --- | --- | --- |
| Age, *M* (*range)* | | 15.69 (1.48) | 15.64 (1.38) |
| Gender, *n* (%) | |  |  |
| Male | | 69 (41%) | 87 (43%) |
| Female | | 98 (59%) | 116 (57%) |
| Prefer not to say | | 0 (0%) | 0 (0%) |
| HSC | | 5.08 (1.60) | 5.76 (1.08) |
| Item 1 | 4.91 (1.91) | 6.03 (1.01) |  |
| Item 2 | 5.11 (1.48) | 5.84 (1.07) |  |
| Item 3 | 5.37 (1.50) | 6.67 (0.48) |  |
| Item 4 | 5.53 (1.58) | 6.19 (0.98) |  |
| Item 5 | 5.63 (1.32) | 6.79 (0.41) |  |
| Item 6 | 4.10 (2.07) | 4.89 (1.78) |  |
| Item 7 | 5.00 (1.69) | 6.30 (0.88) |  |
| Item 8 | 5.26 (1.61) | 6.12 (1.01) |  |
| Item 9 | 5.56 (1.49) | 6.39 (0.77) |  |
| Item 10 | 5.00 (1.83) | 6.10 (1.04) |  |
| Item 11 | 4.78 (1.61) | 5.90 (1.19) |  |
| Item 12 | 21.3 (4.2) | 24.2 (2.5) |  |
| Total sum core | 61 (10) | 73 (5) |  |
| Total mean core | 5.11 (0.82) | 6.08 (0.45) |  |
| AES sum score | 21.3 (4.2) | 24.2 (2.5) |  |
| EOE sum score | 26.0 (5.2) | 31.8 (2.6) |  |
| LST sum score | 14.0 (4.6) | 17.0 (2.7) |  |
| AES mean score | 5.32 (1.06) | 6.05 (0.61) |  |
| EOE mean score | 5.21 (1.04) | 6.36 (0.52) |  |
| LST mean score | 4.67 (1.52) | 5.67 (0.88) |  |

*Notes.* HSC = Highly Sensitive Child Scale. *M* = mean. *N* = sample size. *SD* = standard

deviation.

# Table S12

# *Descriptive Statistics of Classes of the HSC Parent-Report Classes of the Adolescent Group*

|  | | **Green:**  **highly sensitive**  ***n* = 49** | **Blue:**  **Low sensitive**  ***n* = 69** | **Yellow: Emotionally reactive sensitive**  ***n* = 57** | **Red: Sensory sensitive**  ***n* = 75** |
| --- | --- | --- | --- | --- | --- |
| Age, *M* (*range)* | | 17.82 (0.85) | 17.68 (0.94) | 16.65 (1.04) | 17.96 (0.96) |
| Gender, *n* (%) | |  |  |  |  |
| Male | | 25 (51%) | 36 (52%) | 25 (44%) | 36 (48%) |
| Female | | 24 (49%) | 33 (48%) | 32 (56%) | 39 (52%) |
| HSC | | 5.06 (1.70) | 3.86 (1.78) | 5.11 (1.13) | 4.80 (1.42) |
| Item 1 | 5.71 (1.04) | 2.59 (1.20) | 6.37 (0.72) | 3.69 (1.47) |  |
| Item 2 | 5.67 (1.46) | 5.33 (1.18) | 4.33 (1.74) | 6.67 (0.50) |  |
| Item 3 | 6.37 (0.73) | 3.84 (1.54) | 2.89 (1.59) | 4.27 (1.74) |  |
| Item 4 | 6.18 (0.99) | 5.04 (1.38) | 4.44 (1.49) | 6.57 (0.60) |  |
| Item 5 | 6.20 (0.87) | 4.09 (1.53) | 3.58 (1.48) | 4.73 (1.60) |  |
| Item 6 | 3.98 (2.22) | 2.93 (1.69) | 4.60 (1.37) | 2.88 (1.72) |  |
| Item 7 | 5.76 (1.22) | 3.12 (1.38) | 4.70 (1.48) | 3.63 (1.51) |  |
| Item 8 | 5.02 (1.60) | 3.38 (1.52) | 5.86 (1.08) | 3.24 (1.51) |  |
| Item 9 | 6.35 (0.78) | 5.39 (1.44) | 3.16 (1.22) | 6.73 (0.45) |  |
| Item 10 | 5.59 (1.37) | 3.35 (1.42) | 6.12 (0.95) | 3.81 (1.49) |  |
| Item 11 | 6.10 (1.07) | 3.84 (1.37) | 4.09 (1.76) | 4.24 (1.76) |  |
| Item 12 | 23.3 (2.8) | 19.6 (3.4) | 17.0 (3.1) | 24.8 (1.8) |  |
| Total sum core | 68 (6) | 47 (8) | 55 (7) | 55 (6) |  |
| Total mean core | 5.67 (0.50) | 3.90 (0.64) | 4.60 (0.56) | 4.61 (0.52) |  |
| AES sum score | 23.3 (2.8) | 19.6 (3.4) | 17.0 (3.1) | 24.8 (1.8) |  |
| EOE sum score | 29.4 (2.9) | 18.3 (4.5) | 21.1 (4.0) | 20.1 (4.8) |  |
| LST sum score | 15.3 (3.0) | 8.9 (2.7) | 17.1 (1.9) | 10.4 (3.0) |  |
| AES mean score | 5.82 (0.71) | 4.91 (0.84) | 4.26 (0.77) | 6.19 (0.44) |  |
| EOE mean score | 5.89 (0.58) | 3.65 (0.91) | 4.22 (0.80) | 4.02 (0.95) |  |
| LST mean score | 5.10 (1.01) | 2.96 (0.92) | 5.70 (0.62) | 3.46 (1.00) |  |
| *Notes.* HSC = Highly Sensitive Child Scale. *M* = mean. *N* = sample size. *SD* = standard deviation. | | | | | |
